# High RBM3 expression is associated with an improved survival and oxaliplatin response in patients with metastatic colorectal cancer

**DOI:** 10.1371/journal.pone.0182512

**Published:** 2017-08-11

**Authors:** Christina Siesing, Halfdan Sorbye, Anca Dragomir, Per Pfeiffer, Camilla Qvortrup, Fredrik Pontén, Karin Jirström, Bengt Glimelius, Jakob Eberhard

**Affiliations:** 1 Department of Clinical Sciences, Division of Oncology and Pathology, Lund University, Skåne University Hospital, Lund, Sweden; 2 Department of Oncology, Haukeland University Hospital and Department of Clinical Science, University of Bergen, Bergen, Norway; 3 Section of Pathology, Uppsala university Hospital and Department of Immunology, Genetics and Pathology, Uppsala University, Uppsala, Sweden; 4 Department of Oncology, Odense University Hospital, Odense, Denmark; 5 Department of Immunology, Genetics and Pathology, Section of Experimental and Clinical Oncology, Uppsala University, Uppsala, Sweden; Catalan Institute of Oncology, SPAIN

## Abstract

**Background:**

High expression of the RNA-binding motif protein 3 (RBM3) has been shown to correlate, with prolonged survival in several malignant diseases and with the benefit of platinum-based chemotherapy in ovarian cancer. The aim of this study was to evaluate RBM3 in metastatic colorectal cancer (mCRC) as a prognostic factor for overall survival and in relation to benefit of first-line chemotherapy.

**Methods:**

Immunohistochemical staining was conducted and evaluated in tumours from 455 mCRC patients. Kaplan-Meier analysis and Cox regression proportional hazards models were used to access the impact of RBM3 expression on overall survival (OS) and progression-free survival (PFS).

**Results:**

High RBM3 expression, both nuclear and cytoplasmic, was an independent prognostic factor for prolonged OS (hazard ratio [HR] 0.67, 95% confidence interval [CI] 0.50–0.90 and HR 0.66, 95% CI 0.48–0.91, respectively). PFS was significantly longer in patients with high RBM3 expression who had received first-line oxaliplatin based treatment, compared to those who had received irinotecan based treatment, both regarding nuclear and cytoplasmic expression (p-value 0.020 and 0.022 respectively).

**Conclusion:**

High RBM3 expression is an independent predictor of prolonged survival in mCRC patients, in particular in patients treated with first-line oxaliplatin based chemotherapy.

## Introduction

Colorectal cancer (CRC) is the third most common cancer in the world affecting 1.4 million people each year [1]. Approximately 20% have metastatic CRC (mCRC) at the time of diagnosis and about 20% subsequently develop metastatic disease, most with incurable disease [2]. Median survival for mCRC varies between 5–6 months in untreated patients and approaches 30 months in selected groups with good performance status having received optimal treatment [3–6]. Palliative chemotherapy aims to prolong the life of patients and ameliorate quality of life. There is a great need to identify biomarkers that predict response to chemotherapy, in order to save patients from affliction and contribute to better palliation. Chemotherapy for mCRC is based on 5-fluorouracil (5-FU) with the addition of either irinotecan or oxaliplatin. Irinotecan combined with 5-FU, as in FOLFIRI, increases progression-free survival (PFS) and overall survival (OS) [7]. Oxaliplatin combined with 5-FU, as in FOLFOX, increases PFS and likely also OS [7, 8]. Oxaliplatin cross-links the DNA strands and induces apoptosis [9]. Oxaliplatin works synergistically with 5-FU, probably via down regulation of thymidylate synthase [10]. First-line chemotherapy with irinotecan based chemotherapy or oxaliplatin based chemotherapy seem to give a similar overall survival and no data have shown preference for one schedule over the other as first-line treatment [11].

RNA-binding motif protein 3 (RBM3) is an RNA and DNA-binding protein that in response to various types of cellular stress, e.g. hypothermia [12], hypoxia [13], and oxidative stress [14], is required for cell proliferation [12, 15]. Silencing RBM3 decreases the sensitivity to cisplatin in ovarian cancer cell lines [16]. High RBM3 expression has been associated with improved survival in a number of malignancies, e.g. breast cancer, malignant melanoma, uroepithelial cancer, ovarian cancer, colorectal cancer, gastroesophageal cancer, and testicular cancer [16–22].

In CRC, the beneficial prognostic value of high RBM3 expression has been demonstrated in three independent studies [19, 23, 24], mainly including patients with stage II-III disease. To our best knowledge, the relationship between RBM3 expression and response to chemotherapy in mCRC has not yet been reported.

Therefore, this study aimed to evaluate RBM3 as a prognostic factor in mCRC, overall and in relation to the choice of first-line chemotherapy.

## Materials and methods

### Patients

Sorbye et al have previously described the cohort [4, 25] which consists of 798 patients with unresectable mCRC diagnosed in the catchment area of the oncology units at three university hospitals; Odense University Hospital in Denmark, Haukeland University Hospital in Norway and Uppsala University Hospital in Sweden. All patients diagnosed with mCRC between August 2003 and October 2006 were prospectively registered at referral or using regional cancer registers. Written informed consent was obtained from all participants seen at the clinics. The intention of the cohort was to prospectively study different outcomes in an unselected population with mCRC.

Tumour tissue microarrays (TMAs) from mainly the primary tumour (5 cases from metastatic tissue) were constructed from 462 (58%) cases and RBM3 expression could be evaluated in 455 (98%) of these. In the remaining cases, the material was not sufficient to take a core for TMA, containing too much necrotic tissue to evaluate or not available [25]. A flowchart is shown as S1 Fig. The study was approved by the ethical committees in Norway (Regional Committee for medical and health research ethics-REC West 2009/2052), Sweden (Regional Ethical Committee Uppsala 2015/419) and Denmark (The Regional Scientific Ethical Committees for Southern Denmark S-20162000-48).

### Tissue microarray construction

TMAs were generated for protein expression profiling using immunohistochemistry (IHC). IHC staining, slide scanning and evaluation of outcome were performed in accordance with strategies and standards used in the Human Protein Atlas [26, 27]. Original diagnostic tumour blocks were used as donor blocks for the recipient TMA blocks. Representative tumour tissue from each tumour was sampled in duplicate cores of 1 mm in diameter and assembled in the TMAs. A corresponding haematoxylin and eosin stained section was produced from each TMA block to control for quality and presence of tumour cells.

### Antibodies

IHC was performed as previously described [27], in brief, 4 μm sections of the TMA blocks were cut and automated IHC was performed using a Lab Vision Autostainer 480 (Thermo Fisher Scientific). The mouse monoclonal anti-RBM3 antibody (CAB062559 / clone 0296, Atlas Antibodies AB, Stockholm, Sweden, diluted 1:1 000) was used as primary antibody.

### Analysis of immunohistochemical staining

The IHC staining was evaluated by CS and KJ, who were blinded to clinical and outcome data. Interobserver differences were discussed in order to reach consensus. RBM3 expression was evaluated both in the cytoplasm and in the nuclei, whereby the fraction of positive cells was estimated, and the staining intensity denoted as 0 (negative), 1 (mild), 2 (moderate) and 3 (strong). A multiplier of fraction and intensity was constructed regarding nuclear and cytoplasmic expression (nuclear and cytoplasmic score), concluding a score between 0–3.

### Statistical analyses

Chi square test was used to examine the associations between the nuclear and cytoplasmic RBM3 score and clinicopathological characteristics. Kaplan-Meier and log rank test were applied to illustrate differences in OS and PFS according to RBM3 expression. Classification and regression tree (CRT) analysis was applied to estimate the ultimate prognostic cut-off for the nuclear and cytoplasmic RBM3 scores, respectively. A classification based on negative and positive RBM3 expression was also used. Cox regression proportional hazards models were used to access the impact of RBM3 expression on OS and PFS in both univariable and multivariable analyses. PFS was calculated as time from start of first-line treatment until progression or death. A P-value <0.05 was considered statistically significant. All statistical analyses were preformed using IBM SPSS Statistics version 23.

## Results

### RBM3 expression in relation to patient and tumour characteristics

**Fig 1:**
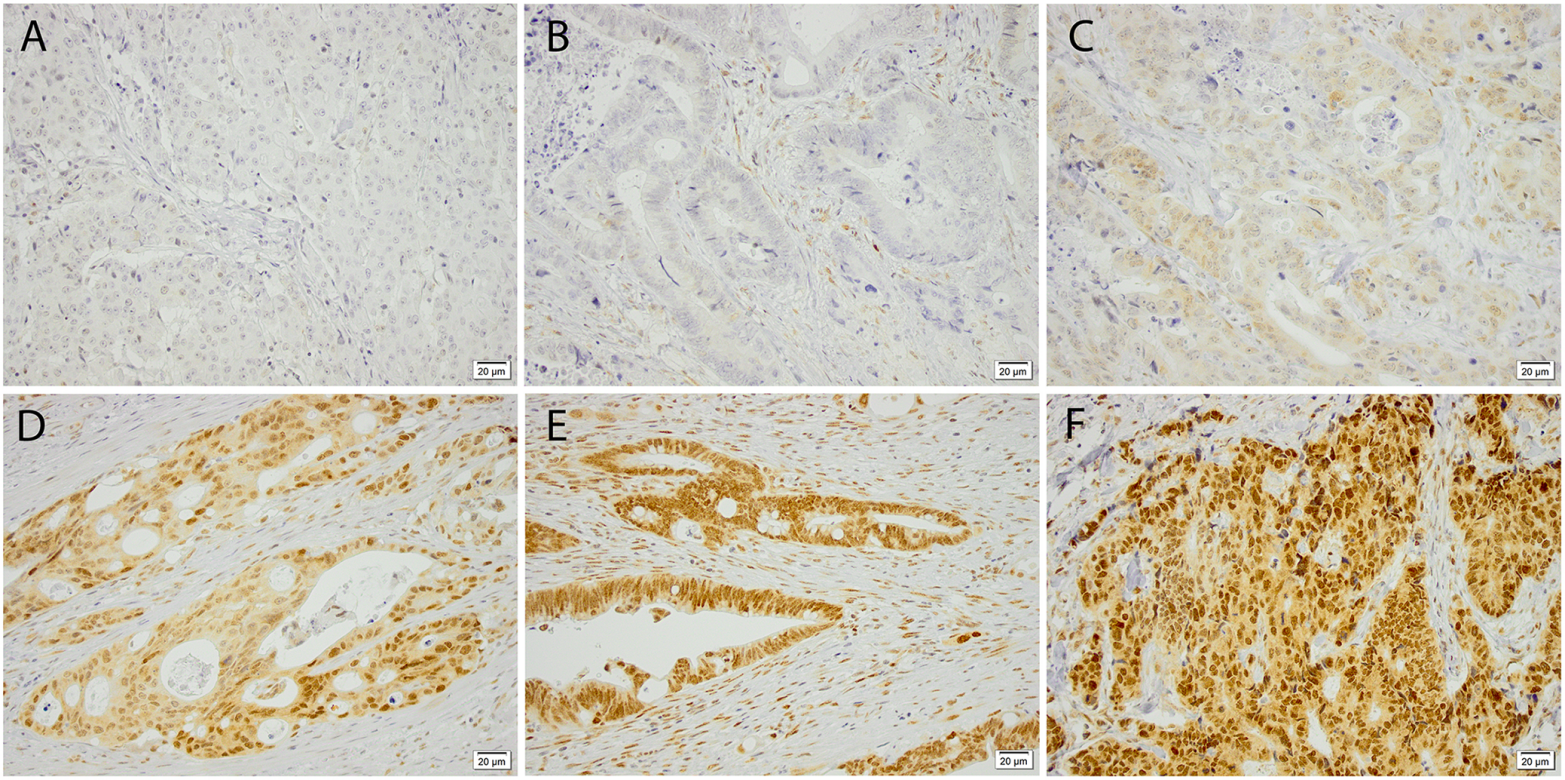
Immunohistochemical images of RBM3 staining. Representing colorectal tumours with (A) negative, (B) weak, (C) moderate and (D-F) strong expression in a varying proportion of tumour cells. All images captured at X 20 magnification.

Among the 455 evaluable cases, 46 (10%) cases were negative for nuclear RBM3 expression and 67 (15%) cases were negative for cytoplasmic RBM3 expression. In the remaining cases, RBM3 was expressed in various intensities and fractions in the nuclei and/or cytoplasm. CRT analysis determined an optimal prognostic cut off at 0.550 for nuclear RBM3 expression and at 0.025 for cytoplasmic RBM3 expression. 166 (36%) cases were denoted as having low and 289 (64%) as having high nuclear expression, and 126 (28%) cases were denoted as having low and 329 (72%) cases as having high cytoplasmic expression, retrospectively. Sample IHC images are shown in Fig 1. Associations of nuclear RBM3 expression with clinicopathological characteristics are shown in Table 1, and for cytoplasmic expression in S1 Table. High nuclear RBM3 expression was statistically significant associated with BRAF wild type tumours, and RBM3 expression was significantly higher in patients who underwent secondary surgery and received irinotecan based treatment. High cytoplasmic RBM3 expression was significantly associated with age <75 years, BRAF wild type tumour, secondary surgery and irinotecan based treatment. Positive nuclear RBM3 expression was significantly more frequent in tumours located in the rectum, in BRAF wildtype tumours and in metachronous disease. Positive cytoplasmic RBM3 expression was significantly more frequent in BRAF wildtype tumors, in patients who had undergone secondary surgery and with metachronous disease.

**Table 1:** Associations between nuclear RBM3 expression and clinicopathological characteristics. ALP; alkaline phosphatase, CEA; carcinoembryonic antigen, KRAS; Kirsten rat sarcoma. (Regarding cytoplasmic expression see S1 Table).

	High Expression	Low expression	*p-value*	Positive	Negative	*p-value*
	N (%)	N (%)	* *	N (%)	N (%)	* *
**Age**						
<75 years	198 (69)	102 (61)	0.126	271 (66)	29 (63)	0.663
≥ 75 years	91 (31)	64 (39)		138 (34)	17 (37)	
**Sex**						
Female	141 (49)	89 (54)	0.322	202 (49)	28 (61)	0.140
Male	148 (51)	77 (46)		207 (51)	18 (39)	
**Performance status **						
0–1	199 (69)	105 (63)	0.222	274 (67)	30 (65)	0.809
>1	90 (31)	61 (37)		135 (33)	16 (35)	
**Primary site**						
Colon	213 (75)	127 (78)	0.352	300 (74)	40 (89)	0.032
Rectum	73 (25)	35 (22)		103 (26)	5 (11)	
Missing	4	3		6	1	
**ALP μg/l**						
Normal	115 (46)	62 (42)	0.503	158 (44)	19 (50)	0.463
Elevated	137 (54)	85 (58)		203 (56)	19 (50)	
Missing	19	37		48	8	
**BRAF**						
Wild-type	234 (83)	117 (72)	0.010	324 (81)	27 (63)	0.005
Mutated	48 (17)	44 (27)		76 (19)	16 (37)	
Missing	7	5		9	3	
**KRAS**						
Wild-type	160 (57)	99 (63)	0.243	232 (58)	27 (66%)	0.349
Mutated	121 (43)	59 (37)		166 (42)	12 (34)	
Missing	8	8		11	7	
**CEA μg/l**						
Normal ≤ 5	46 (28)	22 (25)	0.585	61 (27)	7 (33)	0.502
Elevated > 5	117 (72)	66 (75)		169 (74)	14 (67)	
Missing	126	78		179	25	
**Time for metastasation**						
Synchronous	142 (49)	96 (58)	0.074	207 (51)	31 (67)	0.031
Metachronus	147 (51)	70 (42)		202 (49)	15 (33)	
**Secondary curative surgery**						
Yes	33 (11)	2 (1)	<0.001	34 (8)	1 (2)	0.138
No	256 (89)	163 (99)		374 (92)	45 (98)	
Missing		1		1		
**First line chemotherapy**						
Oxaliplatin-based	113 (39)	53 (32)	0.015	151 (37)	15 (32)	0.132
Irinotecan-based	43 (15)	17 (10)		57 (14)	3 (7)	
5-FU single	27 (9)	30 (18)		47 (11)	10 (22)	
Best Supportive care	88 (31)	56 (34)		129 (32)	15 (32)	
Other	17 (6)	10 (6)		25 (6)	3 (7)	

### RBM3 expression in relation to overall survival

**Fig 2:**
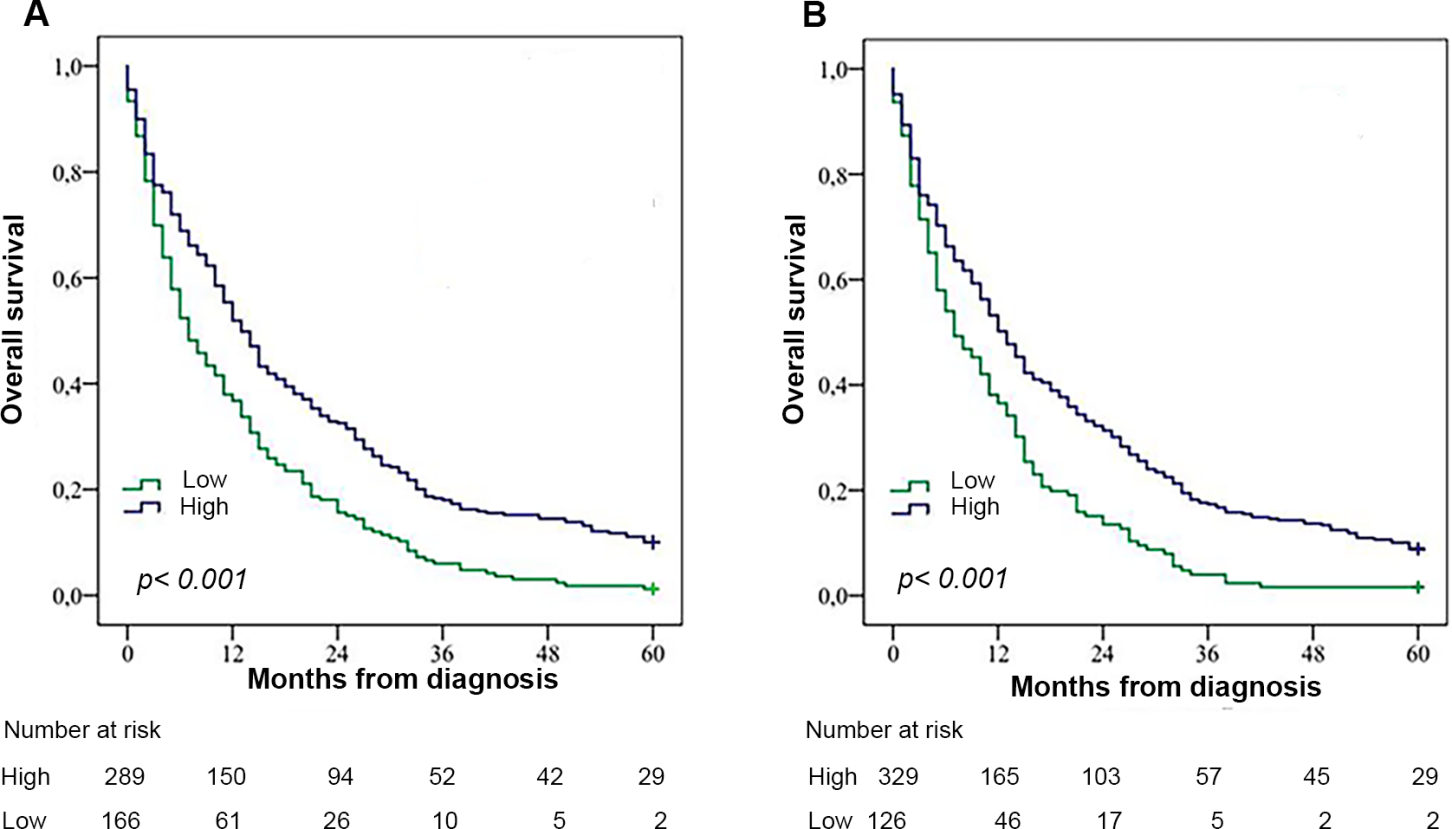
Kaplan-Meier estimates of overall survival. Estimates according to (A) nuclear and (B) cytoplasmic RBM3 expression in the entire cohort.

Kaplan-Meier analyses demonstrated that high RBM3 expression was associated with a significantly longer OS in the entire cohort, both for nuclear (Fig 2A, p<0.001) and cytoplasmic (Fig 2B, p<0.001) expression. This difference remained statistically significant also after exclusion of cases that had received adjuvant treatment (n = 59, data not shown). Median survival was 13 months in the group with high nuclear RBM3 expression and 7 months in the group with low RBM3 expression (p = 0.001). In the group with high cytoplasmic RBM3 expression the median survival was 13 months and 7 months in the group with low cytoplasmic RBM3 expression (p = 0.005). Dichotomisation of RBM3 expression comparing positive to negative expression, revealed a significantly longer OS regarding cytoplasmic expression, but not regarding nuclei expression (S2 Fig).

As shown in Table 2, these associations were confirmed in univariable Cox regression analyses (HR = 0,61, 95% CI = 0,50–0,74 for nuclear and HR = 0,61, 95% CI = 0,49–0,76 for cytoplasmic expression), and remained statistically significant in multivariable analyses (HR = 0.67, 95% CI = 0.50–0.90 for nuclear and HR = 0,66, 95% CI = 0,48–0,91for cytoplasmic expression). When nuclear and cytoplasmic expressions were used as continuous variables statistically significant associations, with a prolonged OS, were seen. The associations only remained statistically significant for nuclear RBM3 expression in a multivariable analysis (Table 2).

**Table 2:** Cox regression analysis of 5-year overall survival according to nuclear and cytoplasmic RBM3 expression. Includes the entire cohort. ALP; alkaline phosphatase, CEA; carcinoembryonic antigen.

	N (events)	Univariable	p	N (events)	Multivariable*	p*
		HR (95%CI)			HR (95%CI)	
**Age**			* *	* *		* *
≥ 75 years	155 (153)	1.00		65 (63)	1.00	
<75 years	300 (271)	0.434 (0.35–0.53)	<0.001	173 (151)	0.723 (0.53–0.99)	0.040
**Sex**			* *	* *		* *
Male	225 (210)	1.00		116 (105)	1.00	
Female	230 (214)	0.95 (0.79–1.15)	0.604	122 (109)	0.68 (0.51–0.90)	0.007
**Performance status **						
0–1	304 (274)	1.00	* *	186 (163)	1.00	* *
>1	151 (150)	3.05 (2.48–3.76)	<0.001	52 (51)	2.06 (1.46–2.90)	<0.001
**Primary site**				* *	* *	* *
Colon	340 (316)	1.00		174 (156)	1.00	* *
Rectum	108 (101)	0.90 (0.72–1.12)	0.339	64 (58)	0.90 (0.64–1.27)	0.554
**ALP μg/l**						
Normal	177 (154)	1.00		111 (92)	1.00	
Elevated	222 (216)	1.69 (1.37–2.09)	<0.001	127 (122)	1.88 (1.37–2.57)	<0.001
**BRAF**						
Wild-type	352 (323)	1.00		195 (171)	1.00	
Mutated	92 (90)	1.72 (1.36–2.18)	<0.001	43 (43)	2.17 (1.48–3.19)	<0.001
**CEA μg/l**						
Normal ≤ 5	68 (57)	1.00		64 (53)	1.00	
Elevated > 5	183 (170)	1.44 (1.07–1.95)	0.017	174 (161)	1.80 (1.28–2.51)	0.001
**Secondary curative surgery **						
Yes	35 (16)	1.00		26 (10)	1.00	
No	419 (407)	6.74 (4.06–11.21)	<0.001	212 (204)	4.97 (2.53–9.76)	<0.001
**First line chemotherapy **						
Oxaliplatin based	166 (142)	1.00		116 (97)	1.00	
Irinotecan based	60 (58)	1.32 (0.97–1.79)	0.078	31 (29)	0.88 (0.57–1.36)	0.571
Other or none	229 (224)	3.00 (2.42–3.73)	<0.001	91 (88)	1.64 (1.05–2.57)	0.031
**Nuclear RBM3 expression **						
Low	166 (164)	1.00		82 (81)	1.00	
High	289 (260)	0.61 (0.50–0.74)	<0.001	156 (133)	0.67 (0.50–0.90)	0.008
**Continuous Nuclear RBM3 expression**	455 (424)	0.74 (0.65–0.83)	<0.001	238 (114)	0.77 (0.65–0.92)	0.005
**Cytoplasmic RBM3 expression **						
Low	126 (124)	1.00		64 (63)	1.00	
High	329 (300)	0.61 (0.49–0.76)	<0.001	174 (151)	0.66 (0.48–0.91)	0.011
**Continuous Cytoplasmic RBM3 expression**	455 (424)	0.71 (0.61–0.84)	<0.001	238 (114)	0.85 (0.68–1.07)	0.161

*The multivariable HRs for other variables than RBM3 are based on inclusion of nuclear RBM3 expression in the model.

### Prognostic value of RBM3 expression in relation to chemotherapy

**Fig 3:**
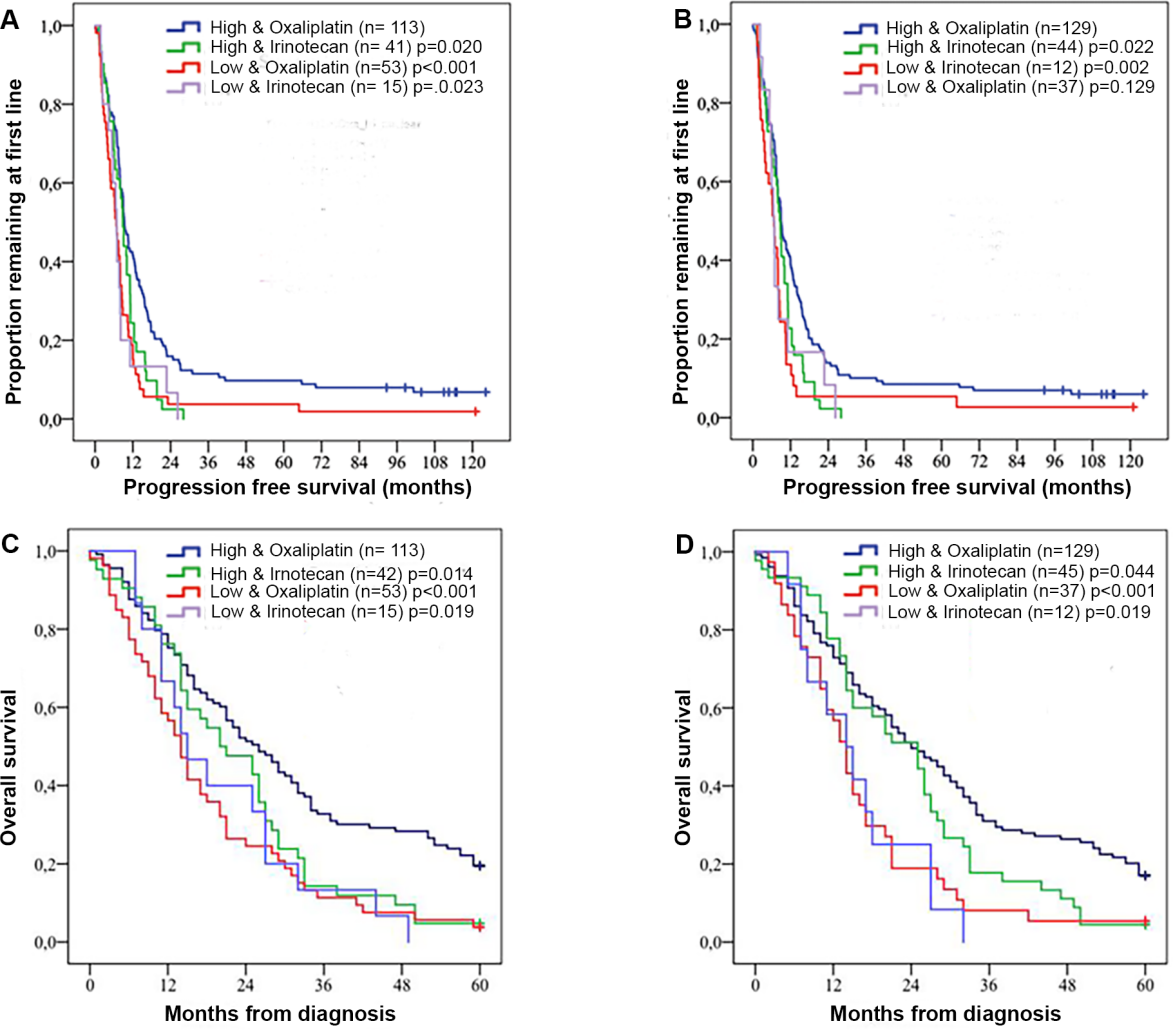
**Kaplan-Meier estimates of progression free (A-B) and overall survival (C-D).** Estimates in combined strata according to high and low RBM3 expression and type of treatment given as first-line. A and C illustrate nuclear expression and B and D cytoplasmic expression. Log rank p-values correspond to pairwise comparison of RBM3 and oxaliplatin treatment with the other strata.

Several associations were seen between RBM3 expression and PFS in strata according to high and low RBM3 expression and type of treatment given as first-line. PFS was longer for patients with tumours displaying high nuclear or cytoplasmic RBM3 expression who received oxaliplatin based treatment as first-line chemotherapy, in comparison to irinotecan based treatment (p-value 0.020 for nuclear expression and 0.022 for cytoplasmic expression). (Fig 3A and 3B) The median PFS was 9.35 months in the group with high nuclear RBM3 expression that received oxaliplatin based chemotherapy and 8.80 months in the group with high nuclear expression that received irinotecan based chemotherapy (p = 0.002). In the group with high cytoplasmic expression the median PFS was 8.99 months in the oxaliplatin group and 8.77 in the irinotecan group (p = 0.028).

Kaplan-Meier analysis of OS revealed that patients with tumours displaying high nuclear or cytoplasmic RBM3 expression who received oxaliplatin based treatment as first-line chemotherapy had significantly longer survival compared to all other strata (Fig 3C and 3D). Especially long-term survival was increased if oxaliplatin based chemotherapy was used as first-line treatment when RBM3 expression was high. This difference remained statistically significant also after exclusion of patients who had received adjuvant treatment (n = 59, data not shown). As seen in S2 Fig, positive RBM3 expression and oxaliplatin based treatment as first-line meant a significantly longer PFS (p = 0.022 for nuclear and p = 0.030 for cytoplasmic expression) compared to irinotecan based treatment.

As shown in Table 3 and S3 Table high nuclear and cytoplasmic expression was associated with complete or partial response to first-line chemotherapy, prolonged median PFS and initiation of second line chemotherapy. Prolonged PFS was the only statistically significant difference between the groups of positive and negative RBM3 expression and was seen regarding cytoplasmic expression.

**Table 3:** Treatment and treatment response. Divided according to high and low nuclear RBM3 expression, and positive and negative nuclear RBM3 expression. (Regarding cytoplasmic expression see S3 Table).

		Nuclear expression RBM3					
	All patients	High expression	Low expression	p-value	Positive	Negative	p-value
	N(%)	N (%)	N (%)		N (%)	N (%)	
All patients	455	289 (64)	166 (36)		409 (90)	46 (10)	
1-line chemotherapy	283 (62)	175 (61)	108 (65)	0.292	255 (62)	28 (61)	0.845
1-line combination oxaliplatin	166 (36)	113 (39)	53 (71)	0.143	151 (30)	15 (32)	0.542
1-line combination irinotecan	57 (13)	42(15)	15 (9)	0.094	54 (13)	3 (7)	0.190
**Response rate 1-line**							
CR/PR	103 (23)	76 (26)	27 (16)	0.015	94 (23)	9 (20)	0.114
PD	45 (10)	24 (8)	21(13)		37 (9)	8 (17)	
PFS 1-line median in months	7.85	8.93	6.08	<0.001	7,95	6.60	0.319
2-line chemotherapy	163 (36)	118 (41)	45 (33)	0.004	148 (36)	15 (33)	0.624
**Response rate 2-line**							
CR/PR	25 (5)	20 (7)	5 (3)	0.208	24 (6)	1 (2)	0.176
PD	56 (12)	37(13)	19 (11)		48 (12)	8 (17)	

CR = complete response, PR = partial response, PD = progressive disease as best response, PFS = progression free survival

There was a statistically significant interaction between RBM3 expression and oxaliplatin based treatment, in relation to PFS (p = 0.047). No such interaction was seen in relation to OS (data not shown).

Notably, there were no significant differences in PFS or OS between patients having received oxaliplatin based treatment and those who had received other types of chemotherapy regardless of RBM3 expression (S3 Fig). Synchronous disease was associated with oxaliplatin based treatment, however no other statistically significant differences regarding patient characteristics between the oxaliplatin treated group and the irinotecan treated group were seen (S2 Table).

## Discussion

Our results demonstrate that high RBM3 expression correlates with longer OS in patients with mCRC. In patients with tumours displaying high RBM3 expression, we have also shown a significantly longer PFS in those treated with first-line oxaliplatin based chemotherapy compared to those treated with first-line irinotecan based chemotherapy, although the actual survival gain was less then one month. The association of high RBM3 expression with OS has previously not been studied in a cohort with only mCRC patients. Melling et al [23] evaluated RBM3 expression in primary CRC (stages I-III), and found an association between high RBM3 expression and longer OS. Hjelm et al [19] studied two different cohorts and only 15% of the patient had stage IV disease. They did however not see any correlation between RBM3 expression and disease stage. Of note, while previous studies in primary CRC found nuclear RBM3 expression to be the strongest prognostic factor [19, 23], the results from the present study demonstrate that not only nuclear, but also cytoplasmic RBM3 expression was significantly associated with prolonged survival. These associations between high RBM3 expression and prolonged oxaliplatin response are in line with previous findings in epithelial ovarian cancer, where a high RBM3 expression was associated with an improved prognosis, and silencing of RBM3 in ovarian cancer cells led to a decreased sensitivity to cisplatin [16].

The optimal prognostic and/or predictive cutoff for RBM3 remains to be established, preferably in prospective studies. Varying cutoffs have been used in previous studies, i.e. week versus strong in the paper by Melling and al[23] and negative versus positive in the paper by Hjelm et al[19]. Our analyses are based on both CRT analyses, and positive versus negative expression, with similar prognostic value, although the former was more significant. The CRT based cutoff appears to well in line with the cutoff used in the study by Melling et al[23].

High nuclear and high cytoplasmic RBM3 expression were associated with BRAF wild type, secondary surgery and irinotecan based treatment. Positive expression, in comparison to negative expression, was related to rectal tumours and BRAF wild type regarding nuclear expression and BRAF wild type and secondary surgery regarding cytoplasmic expression. The association of high RBM3 expression with BRAF wild type tumours is in accordance with earlier reports. Yokota et al [28] among others, have shown that BRAF mutation is associated with shorter OS, which has also been shown in this cohort [25]. Of note, the frequency of BRAF mutation in this cohort is higher compared to prior reports probably due to the non-selected cohort, whereas other reported rates of BRAF mutation often refer to groups that have been included in a clinical trial [25]. The finding of RBM3 expression being significantly higher in cases that had undergone secondary surgery further underpins its association with a less aggressive tumour phenotype, since secondary surgery is relevant when the metastatic burden is limited and the metastases are resectable upfront or after conversion chemotherapy.

All tumours in the herein examined cohort are derived from patients with unresectable mCRC, a group of patients who often have a limited survival. Nevertheless, even in this patient category, high RBM3 expression was demonstrated to be an independent factor of a prolonged survival. It must however be pointed out that the tumour samples examined in this study were mainly obtained from the primary tumours. Moreover, there were no paired specimens from primary tumours and metastases. Therefore, we cannot draw any conclusions on the expression of RBM3 in the metastases, which may differ from the primary tumours. However, since chemotherapy may alter the expression of biomarkers [29, 30], the survival analyses were also performed after exclusion of patients who had received adjuvant chemotherapy, with similar results. A few other studies have compared RBM3 expression in primary tumours and metastases. In malignant melanoma, Jonsson et al [17] reported that RBM3 expression was downregulated in metastases as compared with primary tumours. In contrast, in oesophageal and gastric adenocarcinoma, RBM3 expression was similar in primary tumours and lymph node metastases [22].

Considering this, the number of TMAs available (58%) must be considered adequate. It could be discussed if TMA cores are representative of the whole tumour. To adjust for heterogeneity issues, duplicate 1 mm cores were obtained from different tumour areas. Furthermore, the TMA technique is a good method to demonstrate the connection between molecular alterations and clinical endpoints [31].

It is important to bear in mind that different physicians selected treatment for their patients and we can only speculate on the rationale for selecting a certain treatment. Irinotecan based treatment were, for example, initiated more often to patients that had metachronous disease. However, the difference in OS, regarding both nuclear and cytoplasmic expression, remained significant when adjuvant treated patients were excluded.

Palliative chemotherapy is a balance act between effects and side effects. Peripheral neuropathy is a common side effect of oxaliplatin treatment that may lead to considerable disability in everyday life, for example leading to difficulties using a keyboard or buttoning a shirt. Since the effect of oxaliplatin appears to be limited in patients with tumours displaying low RBM3 expression, several patients could be spared suffering, at least temporarily, if another agent is given in first-line.

In summary, the results demonstrate that high RBM3 expression is associated with prolonged survival in patients with mCRC, in particular in those having received first-line oxaliplatin based chemotherapy. These findings merit further study, since they are of potential clinical relevance. It would also be of interest to explore the impact of RBM3 expression on oxaliplatin sensitivity in CRC cells *in vitro*.

## Supporting information

S1 Table Associations between cytoplasmic RBM3 expression and clinicopathological characteristics. ALP; alkaline phosphatase, CEA; carcinoembryonic antigen, KRAS; Kirsten rat sarcoma.
(XLSX)

S2 Table Patient characteristics regarding type of chemotherapy given as first-line. CEA; carcinoembryonic antigen.
(XLSX)

S3 Table Treatment and treatment response divided according to high and low cytoplasmic RBM3 expression and positive and negative cytoplasmic RBM3 expression. CR = complete response, PR = partial response, PD = progressive disease.
(XLSX)

S1 Fig A flowchart outlining cases available for tissue microarray construction and analysis of RBM3 expression within the entire cohort of 798 cases. (TIF)

S2 Fig Kaplan-Meier estimates of overall survival according to (A) nuclear and (B) cytoplasmic RBM3 expression.
(TIF)

S3 Fig Kaplan-Meier estimates of (A) progression free survival and (B) overall survival according to chemotherapy regimen given as first-line.
(TIF)
