# Supplementary figures and images for: High RBM3 expression is associated with an improved survival and oxaliplatin response in patients with metastatic colorectal cancer
Source: PLoS One. 2017 Aug 11;12(8):e0182512. doi: 10.1371/journal.pone.0182512 (PMC5553773; doi:10.1371/journal.pone.0182512)

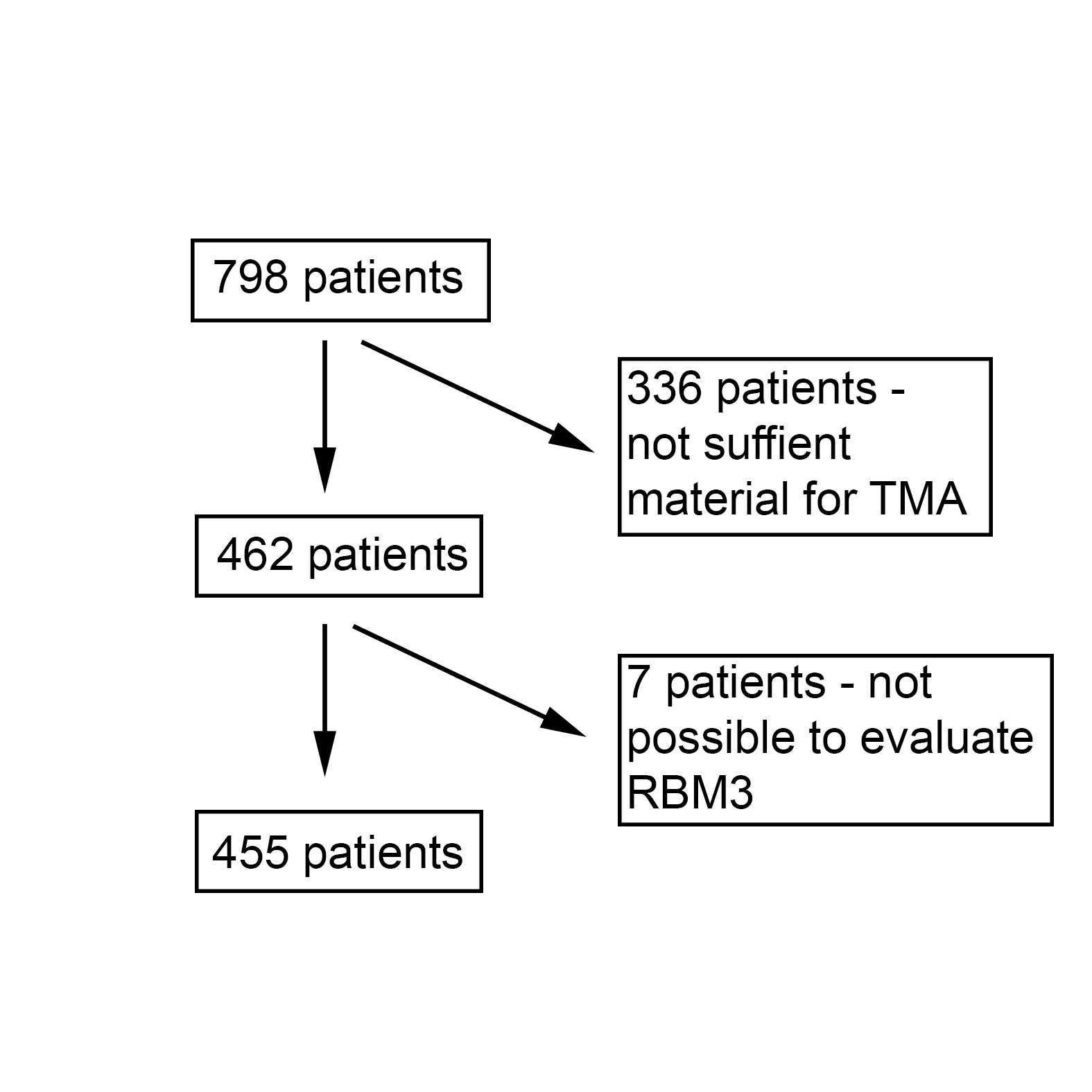

Supplement: S1 Fig — (TIF) [file pone.0182512.s004.tif]

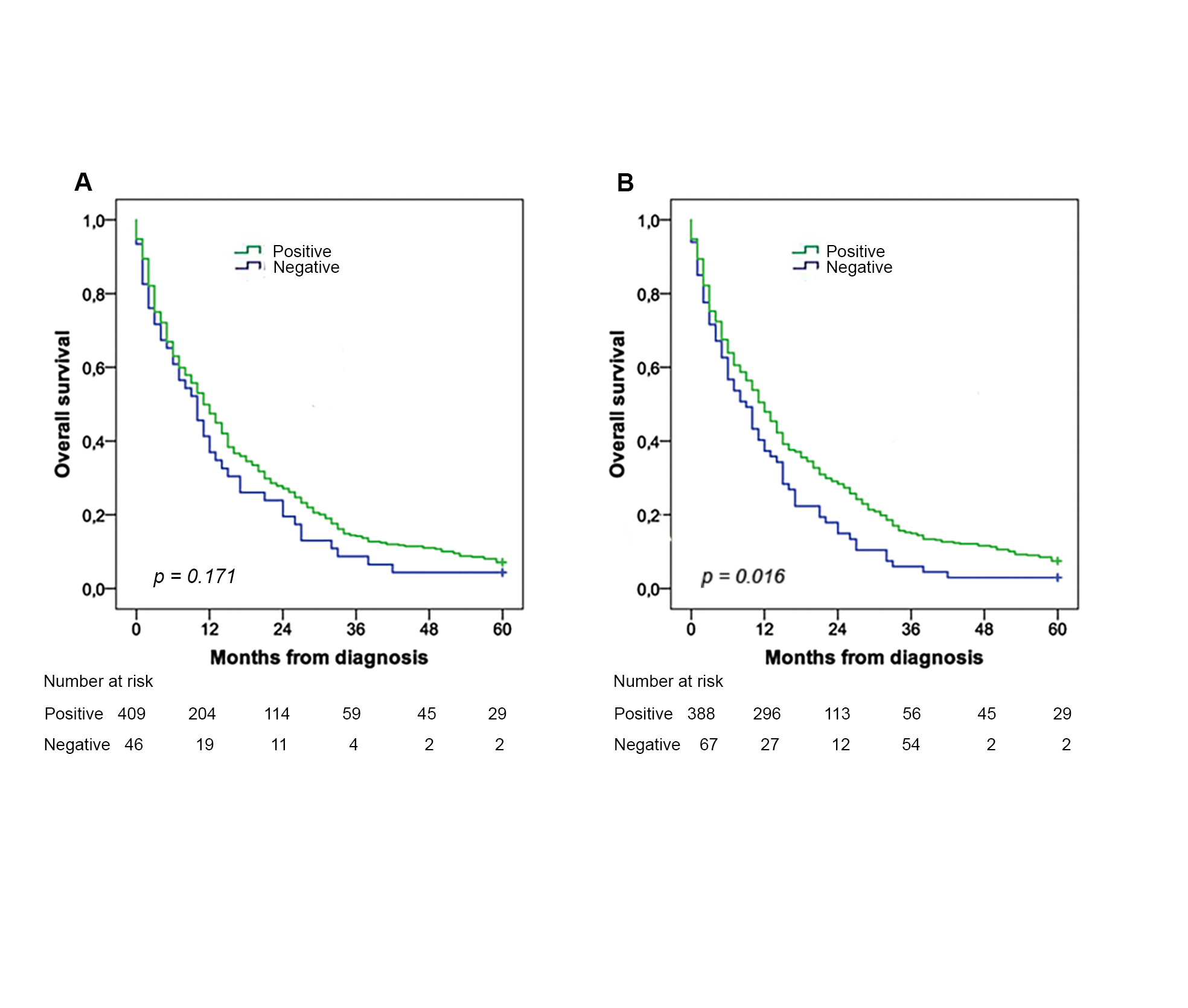

Supplement: S2 Fig — Kaplan-Meier estimates of overall survival according to (A) nuclear and (B) cytoplasmic RBM3 expression. (TIF) [file pone.0182512.s005.tif]

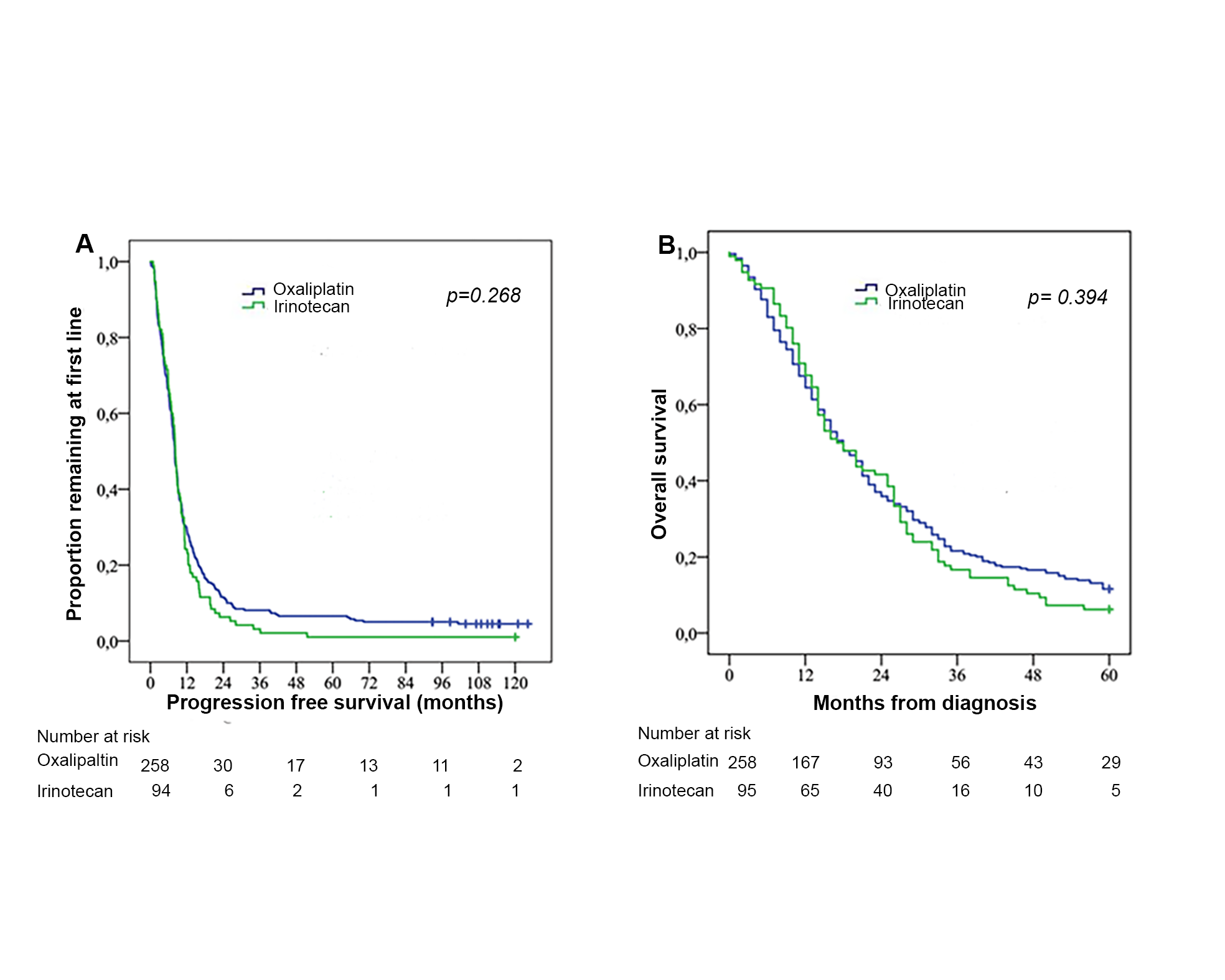

Supplement: S3 Fig — Kaplan-Meier estimates of (A) progression free survival and (B) overall survival according to chemotherapy regimen given as first-line. (TIF) [file pone.0182512.s006.tif]
